# Preliminary evidence for genetic overlap between body mass index and striatal reward response

**DOI:** 10.1038/s41398-017-0068-4

**Published:** 2018-01-10

**Authors:** T. M. Lancaster, I. Ihssen, L. M. Brindley, D. E. Linden

**Affiliations:** 1 Neuroscience and Mental Health Research Institute, Cardiff University, Cardiff, UK; 2 Cardiff University Brain Research Imaging Centre, School of Psychology, Cardiff University, Cardiff, UK; 3 MRC Centre for Neuropsychiatric Genetics and Genomics, Institute of Psychological Medicine and Clinical Neurosciences, Cardiff School of Medicine, Cardiff University, Cardiff, UK; 4 Department of Psychology, Queen’s Campus, Durham University, Durham, UK

## Abstract

The reward-processing network is implicated in the aetiology of obesity. Several lines of evidence suggest obesity-linked genetic risk loci (such as *DRD2* and *FTO*) may influence individual variation in body mass index (BMI) through neuropsychological processes reflected in alterations in activation of the striatum during reward processing. However, no study has tested the broader hypotheses that (a) the relationship between BMI and reward-related brain activation (measured through the blood oxygenation-dependent (BOLD) signal) may be observed in a large population study and (b) the overall genetic architecture of these phenotypes overlap, an assumption critical for the progression of imaging genetic studies in obesity research. Using data from the Human Connectome Project (*N* = 1055 healthy, young individuals: average BMI = 26.4), we first establish a phenotypic relationship between BMI and ventral striatal (VS) BOLD during the processing of rewarding (monetary) stimuli (*β* = 0.44, *P* = 0.013), accounting for potential confounds. BMI and VS BOLD were both significantly influenced by additive genetic factors (H2r = 0.57; 0.12, respectively). Further decomposition of this variance suggested that the relationship was driven by shared genetic (*ρ*_g_ = 0.47, *P* = 0.011), but not environmental (*ρ*_E_ = −0.07, *P* = 0.29) factors. To validate the assumption of genetic pleiotropy between BMI and VS BOLD, we further show that polygenic risk for higher BMI is also associated with increased VS BOLD response to appetitive stimuli (calorically high food images), in an independent sample (*N* = 81; *P*_FWE−ROI_ < 0.005). Together, these observations suggest that the genetic factors link risk to obesity to alterations within key nodes of the brain's reward circuity. These observations provide a basis for future work exploring the mechanistic role of genetic loci that confer risk for obesity using the imaging genetics approach.

## Introduction

Genome-wide association studies (GWAS) demonstrate that obesity (as measured via body mass index; BMI) has a complex polygenic architecture where a large number of common risk alleles are likely to confer susceptibility^1,2^. However, the mechanisms by which these loci confer risk are largely unknown. Neuroimaging studies provide evidence that individuals with higher BMI have alterations in the processing of hedonic stimuli such as calorific food images^3–5^ and monetary reward^6–8^. Individuals at high risk for obesity also show a similar neural phenotype, suggesting that the altered reward response may be a neural antecedent to weight gain^9^. Using functional magnetic resonance imaging (fMRI), studies have also begun to elucidate mechanistic roles for candidate obesity risk loci (such as loci within *DRD2, FTO*) in the reward circuitry of the human brain^10–13^. These studies suggest that obesity risk loci may alter eating behaviour via the regulation of key reward-processing nodes such as the striatum^14,15^.

However, under a polygenic model of obesity^16,17^, single genetic risk factors (such as loci within/near to *FTO, DRD2*) are likely to exert modest influence over BMI and associated putative neural risk mechanisms such as altered brain networks^18,19^. This makes it difficult to gain adequate power to detect the effects of single obesity risk loci in small populations, which may hinder progress towards therapeutic and intervention strategies. In the current study, we aim to test the broader hypothesis of polygenic pleiotropy between BMI and the neural response to reward. Elucidating the contribution of these potential causal factors (e.g., risk genes) is essential for understanding the neurobiological mechanisms by which risk for obesity is conferred (and ultimately for the appropriate targeting of interventions in at-risk populations).

The present investigation therefore aims to explore the genetic relationship between BMI and reward-related function (blood oxygen level dependency (BOLD)) in a well-powered and deeply phenotyped multimodal genetic neuroimaging consortium (http://www.humanconnectome.org/). As obesity is associated with altered BOLD during monetary rewards as well as appetitive stimuli, we anticipate that the BOLD response for rewarding stimuli will be linked to BMI^5–7^. We choose to restrict our neural response phenotype to BOLD within the ventral striatum (VS), as it has been previously demonstrated to be robustly activated during the Gambling paradigm acquired as part of the Human Connectome Project (HCP)^20^. In the HCP data, we first aim to demonstrate an association between BMI and the striatal reward response. This will build on previous associations between BMI and striatal BOLD in response to monetary rewarding stimuli^5,6^. We then aim to estimate the heritability of BMI and the VS BOLD responses. Lastly, we exploit the kinship structure (the twin pairs) within the HCP consortium in a bivariate correlation analysis to decompose the putative phenotypic association into shared genetic and/or environmental influence. We anticipate that a potential association between BMI and VS BOLD may be explained by genetic and/or environmental factors. Together, these analyses aim to (a) establish and (b) decompose phenotypic associations between BMI and system-level alterations in the brain's reward system into genetic and/or environmental influences. Any notable sources of phenotypic covariance (e.g., additive genetic and environmental) may be useful in informing mechanisms that link BMI and the reward circuitry. In an independent genetic neuroimaging sample we also aim to validate potential (genetic) pleiotropy between BMI and VS BOLD. In this study, we explore the putative genetic relationship using a risk profile score (RPS) approach to index the impact of BMI-related risk alleles on the VS BOLD during the processing of appetitive food. In this analysis we anticipated a positive relationship between BMI-RPS and VS BOLD in response to appetitive stimuli. Together, these analyses will decompose the causal (genetic) mechanisms that may underpin the association between alterations in BMI and responsiveness to rewarding stimuli.

## Materials and methods

### Participants

#### HCP sample

Participants were drawn from the March 2017 public data release from the HCP (*N* = 1200). All participants were aged from 22 to 35, for all inclusion/exclusion criteria see Van Essen et al^21^. Briefly, the study excluded individuals with a history of psychiatric disorder, substance abuse, neurological or cardiovascular disease and associated hospitalisation or long-term (>12 months) pharmacological/behavioural treatment. BMI was measured as self-reported weight (kg) divided by self-reported height (cm) squared. Participants were excluded from the current analyses if they lacked good-quality structural magnetic resonance imaging data, or had missing relevant interview/questionnaire data (Table 1; for demographic details of each analysis). The overall sample size, including non-related individuals, was *N* = 1055, which has over 90% power to detect a small effect (*R* ^2^ = 0.1). For further information on the HCP pedigree/kinship structure see http://www.humanconnectome.org/storage/app/media/documentation/s1200/HCP_S1200_Release_Reference_Manual.pdf.

**Table 1:** Demographics for both samples

Sample	MZ/DZ pairs	*N* (all)	Mean FD (±SD)	Age (±SD)	Sex (M/F)	BMI (±SD)
HCP	126/72	1055	0.086 ± (0.033)	28.77 ± 3.69	483/572	26.44 ± 5.11
Cardiff	n/a	81	0.083 ± (0.055)	23.9 ± 3.55	32/49	n/a

Descriptive statistics for the HCP sample were calculated from the complete sample, used in the linear mixed model regression

MZ/DZ twin pairs represent the complete number of twin pairs used in all the univariate and bivariate correlations for BMI and VS BOLD, controlling for all covariates

#### Cardiff sample

One hundred right-handed Caucasian (of western European descent) volunteers aged 19–47 were recruited from Cardiff University (staff and/or students) for a study involving several MRI, MEG and behavioural paradigms. No participants reported any psychiatric illness^22^ or use of psychotropic medication. Informed consent was obtained for all individuals prior to the study, which was approved by the ethics committee of the School of Psychology, Cardiff University (EC.12.01.10.3071). A sample of *N* = 81 (appetitive picture viewing) participants were included in the final sample after removing individuals with failed quality control of genetic data (*n* = 10) or incomplete imaging data (*n* = 9).

### DNA extraction, genotyping and generation of BMI RPSs

#### Cardiff sample

Genomic DNA was obtained from saliva using Oragene OG-500 saliva kits. Genotyping was performed using custom genotyping arrays (Illumina HumanCoreExome-24 BeadChip) that contain 570,038 genetic variants (Illumina Inc., San Diego, CA). Quality control was implemented in PLINK^23^ to ensure that genotypes did not display ambiguous sex, cryptic relatedness up to third degree relatives by identity of descent or genotyping completeness <97%. We also removed non-European ethnicity admixture detected as outliers in iterative EIGENSTRAT analyses of an LD-pruned data set^24^. SNPs were excluded where the minor allele frequency (MAF) was <1%, if the call rate <98% or if the *χ* ^2^-test for Hardy–Weinberg Equilibrium had a *P* value <1e−04. BMI-RPS was calculated using the method described by the International Schizophrenia Consortium^25^. BMI genetic risk was estimated using publicly available results' data from an international GWAS^2^. Briefly, SNPs (single-nucleotide polymorphisms) were removed from the BMI GWAS data if they had a low (MAF < 0.01), and were subsequently pruned for linkage disequilibrium (*R* ^2^ < 0.2). As SNPs may be correlated, pruning the SNPs ensured that all SNPs included in each BMI-RPS model were fairly independent. BMI-RPS were estimated using the ‘score′ command in PLINK. For each individual, the ‘score′ command averages the number of risk alleles for each BMI-increasing SNP (provided by the independent BMI GWAS summary statistics) and weights each allele by the size of the effect (coefficient) for the allele, as estimated in the BMI GWAS. For our analysis, we restricted the BMI-RPS to SNPs in the GWAS that were nominally associated with BMI (i.e., BMI-RPS *P* threshold (*P*_T_ < 0.05)), a BMI-RPS threshold shown to capture substantial variance in BMI in a large independent sample^26,27^. The BMI-RPS was normally distributed (Shapiro test = 0.53) in our sample.

### Data acquisition

#### HCP sample

Images were acquired using a customised Siemens Skyra 3-T scanner with a 32-channel head coil. For details on data acquisition and preprocessing, see Glasser et al.^28^.

#### Cardiff sample

Gradient echoplanar imaging data were acquired for each subject using a 3 T GT HDx system with an eight-channel receiver at CUBRIC (Cardiff University Brain Research Imaging Centre), School of Psychology, Cardiff University (parameters: 35 slices, slice thickness: 3 mm/1 mm gap; acquisition matrix: 64 × 64; field of view (FOV): 220 mm; repetition time (TR): 2000 ms; echo time (TE): 35 ms; flip angle: 90°; acceleration (ASSET) factor: 2). High-resolution three-dimensional T1-weighted images were also acquired using a three-dimensional fast spoiled gradient echo sequence with 172 contiguous sagittal slices of 1 mm thickness (TR: 7.9 s; TE: 3.0 ms; inversion time (TI): 450 ms; flip angle: 20°; FOV: 256 × 256 × 176 mm; matrix size: 256 × 256 × 192 to yield 1 mm isotropic voxel resolution images). All functional images were first motion-scrubbed, where TRs with a framewise displacement > 0.9 were removed, as previously recommended^29^.

### Description of fMRI paradigms

#### HCP sample (incentive processing)

Reward-related BOLD signal was measured with fMRI during a card-guessing gambling task played for monetary reward, as previously described^30,31^. Briefly, participants completed a card-guessing game where they are required to guess the number (ranging from 1 to 9) on a mystery card in order to win or lose money. Participants were instructed to guess if the mystery card number was more or less than 5 by pressing one of two buttons on the response box. Feedback was provided as the revealed card number and a cue to inform the participant if they received a monetary reward, loss or neutral (no reward/loss; for number 5) trial. The task was presented in blocks of eight trials that were either mostly reward (six reward trials pseudo randomly interleaved with neutral and/or loss trials) or mostly loss (six loss trials interleaved with reward and/or loss trials). For each of the two runs, there were two mostly reward and two mostly loss blocks, interleaved with four fixation blocks (15 s each). Although the participants gambled for potential monetary reward, all participants are rewarded with a standard amount of money during the task.

#### Cardiff sample (appetitive picture viewing)

Participants viewed appetitive food images and neutral stimuli taken from the International Affective Picture System (IAPS)^32^ or Internet resources. We included 18 neutral IAPS pictures having a mean normative valence rating of 4.87 (1 = very unpleasant and 9 = very pleasant) and mean arousal rating of 2.62 (1 = low-arousing and 9 = high-arousing) and nine positive IAPS pictures having a mean normative valence rating of 6.99 with a mean arousal rating of 4.58. Images taken from other resources had been used and validated in a previous study^11^. Picture categories were comparable with regard to semantic homogeneity and perceptual complexity. Neutral pictures showed household objects and positive images depicted appetitive food. Each block lasted 8 s, in which an array consisting of either four random positive or four random neutral images were presented at a rate of 2 s per image. This process was repeated 10 times for each participant. To keep individuals engaged in the task, we included a 1-back monitoring task in which participants had to confirm with a button press each time an image was presented twice in a row within a trial block. For each participant, we embedded four picture repetitions at random positions within the entire sequence of picture-viewing blocks. The number of picture repetitions was balanced across picture categories. There were four picture repetitions for each participant, with an equal number of repetitions occurring for each picture category. Participants viewed a total of 40 stimuli per condition. Inter stimulus intervals were randomly jittered (6–10 s) in order to sample the hemodynamic response at different time points.

### BOLD parameter estimate acquisition

#### HCP sample

Individual, pre-processed task-fMRI (tfMRI) directories for the gambling task were downloaded from the WU-Minn HCP Data—1200 Subjects + 7T data release at https://db.humanconnectome.org/, package type = MSM-Sulc-+MSM-All. For preprocessing steps and preliminary analysis, see ref. ^30^. Briefly, the HCP ‘fMRIVolume' pipeline performs gradient unwarping, motion correction, fieldmap unwarping and grand mean intensity normalisation on the four-dimensional (4D) time series. These volumes are segmented (Brain Boundary Registration), registered to the T1 anatomical volume using nonlinear transformation (FNIRT) and warped to standard (MNI152) space. Parameter estimates were estimated for a pre-processed time series using a general linear model (GLM) using FMRIB's improved linear model (FILM) with autocorrelation correction. Predictors (described in Methods: Incentive Processing Paradigm) were convolved with a double gamma canonical hemodynamic response function to generate regressors. Temporal derivatives of each regressor were added to the GLM as covariates of no interest. Parameter estimates (BOLD) for the contrast (reward > punishment; cope6.feat) were available for 1082 individuals. We chose this contrast to establish potential relationships specifically with reward, rather than punishment processing in the VS^20^. As the paradigm was a card-guessing task, the contrast models reward receipt but did not include an anticipation phase like other paradigms such as the monetary incentive delay task^33,34^. Using the ‘wb_command′ from the connectome-workbench (https://www.humanconnectome.org/software/connectome-workbench.html), we then extracted BOLD parameter estimates from individual subject-pre-processed data (cope6.feat; reward > punishment) for the bilateral nucleus accumbens (VS) as defined by the Harvard-Oxford Subcortical Structural Atlas.

#### Cardiff sample

Image-processing and statistical analyses were conducted using statistical parametric mapping methods as implemented in FMRI Expert Analysis Tool (FEAT, Version 5.98, part of FMRIB's Software Library, www.fmrib.ox.ac.uk/fsl). The following pre-statistics processing was applied: motion correction using MCFLIRT^35^; slice-timing correction using Fourier-space time series phase-shifting; non-brain removal using Brain Extraction Tool^36^; spatial smoothing using a Gaussian kernel of full width half maximum (FWHM) 5 mm; grand mean intensity normalisation of the entire 4D data set by a single multiplicative factor; and high-pass temporal filtering (Gaussian-weighted least-squares straight line fitting, with sigma = 50.0 s). Registration to high-resolution structural (single-subject GLM) and standard space (group-level GLM) images was carried out using FLIRT^35^. Time series analysis was carried out using FILM with local autocorrelation correction^37^. Group-level analysis was carried out using FLAME (FMRIB′s Local Analysis of Mixed Effects)^38^. To index neural responses to positive emotional stimuli in experiment 1, BOLD signal changes were regressed by task predictor functions (positively affective stimuli > neutral stimuli) convolved with a canonical hemodynamic response function.

### VS BOLD quality control

#### HCP sample

Outliers (*N* = 24) were removed from the bilateral striatal BOLD parameter estimates using the interquartile range (IQR) outlier labelling rule (1.5 × IQR (Q3–Q1)) as previously described^39^. After the removal of statistical outliers, VS BOLD was normally distributed (Shapiro test, *P* > 0.05).

### Statistical inferences

#### HCP sample

*Linear mixed modelling*: We first aimed to explore the average relationship between BMI and the VS BOLD across the whole sample (*N* = 1055). On the basis of prior recommendations^40^, we first employed linear mixed-effects models, estimated in R (https://www.r-project.org/) using the *lme4* and *lmeTest* packages^41,42^. BMI was entered into the model as the independent variable with age, sex, education level, height and handedness and head motion (FD_FSL_) as potential confounds. To account for kinship, family structure (Family ID) and zygosity (monozygotic twins, dizygotic and unrelated individuals; coded as a percent DNA shared; 1, 0.5, 0, respectively) were entered into each model as random effects, which under the model assumptions could be freely correlated with each other^40^. We assumed independence between these random slopes to control for potential genetic (as assayed by the random effect of zygosity) and familial environmental (as measured by kinship) correlations. These random effects were modelled to control for potential genetic influence over the phenotypic relationship between BMI and VS BOLD—which we formally explore in the next section. Regression diagnostics complied with assumptions; normal distribution of residuals (Shapiro test: *P* = 0.23) and non-independence of errors (autocorrelation tests performed with Durbin–Watson Statistic: 2.007) and was taken forward for interpretation.

#### HCP sample

*Heritability and co-heritability of BMI and VS BOLD:* heritability and co-heritability for BMI and VS BOLD were estimated using SOLAR (Sequential Oligogenic Linkage Analysis Routines: http://solar.txbiomedgenetics.org ^43^). SOLAR adopts maximum likelihood variance component methods to analyse family-based quantitative data by partitioning the observed covariance into genetic and environmental components, as a function of genetic proximity^43,44^. Pedigree information was calculated using publically available tools for HCP data (https://brainder.org/2016/08/01/three-hcp-utilities/). Heritability (H2r) is defined as the proportion of total phenotypic variance explained by additive genetic factors. The shared genetic variance between BMI and VS BOLD was calculated using bivariate genetic correlation analysis methods, also implemented in SOLAR. Bivariate genetic correlation analysis is performed to calculate the proportion of common genetic variance that influences both BMI and VS BOLD. If the genetic correlation coefficient (*ρ*G) is significantly different from zero, then a significant portion of the variability in the two traits is considered to be influenced by shared genetic factors^45^.

#### Cardiff sample

We ran multiple regression using the combined first-level contrasts (appetitive food image > neutral pictures) for each subject co-varying for BMI-RPS and potential confounds (age and sex). We explored the (a) group-level contrasts (one-sample *t*-tests) and (b) BMI-RPS effects (multiple regression) in the VS region of interest, defined as the bilateral accumbens in the Harvard-Oxford Subcortical Structural Atlas. The family-wise error rate was controlled in all cases with nonparametric permutation testing (5000 permutations) and threshold-free cluster enhancement that effectively controls for multiple comparisons, compared to cluster extent thresholding^46^.

### Head motion confounds

#### HCP sample

As previously reported, there are considerable phenotypic and genetic correlations between BMI and head motion during resting-state fMRI^44^, suggesting that the same genetic variation contributes to both traits. To control for putative confounding effects of head motion on the relationship between BMI and VS BOLD in the HCP data, we used estimations of framewise displacement (Movement_RelativeRMS_mean.txt) for the two tfMRI gambling runs and included the log-transformed mean of the two runs in all phenotypic and genetic analyses.

#### Cardiff sample

To further correct for any potential movement confounds in the Cardiff Sample, motion regressors estimated via MCFLIRT and scrubbed TRs were added as covariates of no interest to the first-level design matrix.

## Results

### Head motion and BMI

#### HCP sample

Consistent with previous reports^44^, we observed phenotypic and genetic correlations between head motion (FD_(FSL)_) and BMI (*ρ*_P_ = 0.63, *ρ*_g_ = 0.78, respectively). We therefore included the log-transformed mean FD_(FSL)_ as a covariate in all univariate and bivariate analyses.

### Linear mixed modelling

#### HCP sample

After quality control and diagnostics, BMI was regressed against the bilateral VS BOLD phenotype. After controlling for fixed effects (covariates) and familial confounds (random effects of familial environmental and genetic correlations), we identified a positive association between BMI and VS BOLD (*β* = 0.44 ± 0.172; *t*_954.4_ = 2.469, *P* = 0.0128). This association was robust to socioeconomic status (employment, relationship status and income).

### Heritability and co-heritability between BMI and VS BOLD

#### HCP sample

We then proceeded to decompose the observed phenotypic relationship between BMI and VS BOLD, in order to establish whether familial genetic and/or environmental factors contributed to the association. Both BMI and VS BOLD were significantly heritable (BMI H2r = 0.57; VS BOLD H2r = 0.12), controlling for age, sex, years of education, height and head motion (Table 2 for statistics). The bivariate analysis in Solar also demonstrated a positive phenotypic relationship between BMI and VS BOLD (*ρ*_P_ = 0.08, *P* = 0.012), controlling for the same covariates. Further decomposition of the variance suggested that BMI and VS BOLD had a shared genetic aetiology (*ρ*_g_ = 0.47 ± 0.21, *P* = 0.011). There was no evidence for shared environmental aetiology (*ρ*_e_ = −0.07 ± 0.06, *P* = 0.29). All univariate and bivariate correlations were also retained when controlling for socioeconomic status (employment, relationship status and income).

**Table 2:** Heritability of traits in the HCP data (twin data)

Phenotype	H2	H2rSE	*P*
Head motion	0.30	0.064	<0.001
BMI	0.57	0.056	<0.001
VS BOLD	0.12	0.062	0.023

*H2r* additive genetic variance for each IDP. *H2rSE* standard error of heritability estimate. All analyses remained significant before/after controlling for covariates. *BOLD* parameter estimates, extracted from native masks from pre-processed single-subject tfMRI_GAMBLING_hp200_s2_level2_MSMAll.feat/GrayordinatesStats/cope6.feat data

### BMI-RPS regression

#### Cardiff sample

**Fig. 1:**
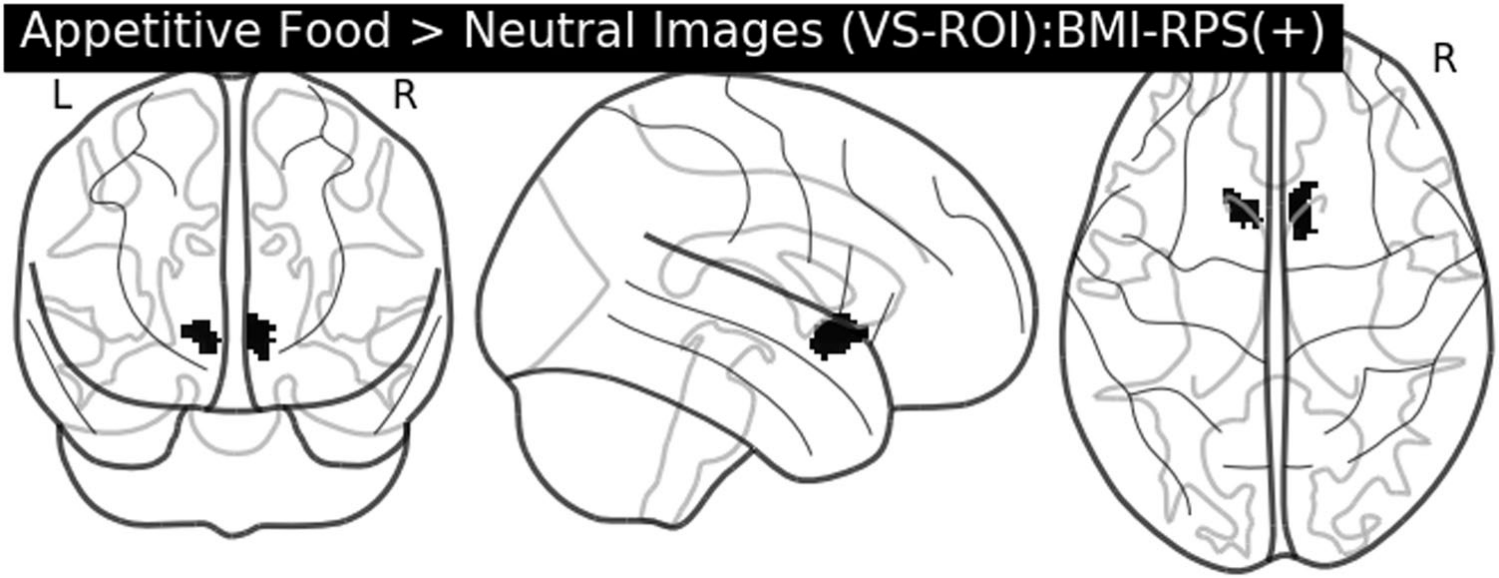
Positive association between BMI-RPS and VS BOLD in the appetitive food > neutral stimuli contrast in the Cardiff sample (*N* = 81). Image is (1−*P* value) map, where all active voxels (in black) are voxels that survive the family-wise error correction (*P*_FWE-ROI-corrected_ < 0.05) across the VS using threshold-free cluster enhancement (TFCE)

While we did not have a BMI measure for the Cardiff sample, the BMI-RPS was positively associated with sex-adjusted weight (kg) in the sample (*t*_1,79_ = 2.362, *P* = 0.021), supporting the validity of the BMI-RPS approach. A one-sample *t*-test (appetitive food > neutral images) showed a significant recruitment of the bilateral VS as previously described^47^. Crucially, there was a significant positive association between BMI-RPS and BOLD in clusters within the right (*k* = 123, *P*_FWE−ROI_ = 0.005 [*x* = 10, *y* = 10, *z* = −6)) and left (*k *= 77, *P*_FWE−ROI_ = 0.017 [*x* = −10, *y* = 12, *z* = −4)) VS (Fig. 1). There were no significant associations between BMI-RPS and BOLD across the whole brain or negative associations across the whole brain or within the VS (*P* > 0.1 in all cases). This relationship between BMI-RPS and VS BOLD remained after controlling for sex-adjusted weight (*P*_FWE−ROI_ = 0.013; *P*_FWE−ROI_ = 0.034). The direction of the association between VS BOLD and sex-adjusted weight was positive as expected, but not significant (*P*_FWE−ROI_ = 0.16). This association was attenuated when BMI-RPS was added into the model (*P*_FWE-ROI_ = 0.45).

## Discussion

We first establish a positive relationship between BMI and striatal activation in a large sample of healthy individuals. While previous studies have shown genetic links between BMI and structural imaging measures (such as reduced grey matter volume in orbitofrontal areas^48^), ours is the first large study to demonstrate an association between BMI and BOLD. We also established that BMI and reward-dependent striatal activation were heritable traits. While several lines of evidence show that additive genetic factors contribute to adiposity^2^, we suggest that this study is the first to show evidence for additive genetic factors in VS BOLD during a gambling task, although there are previous accounts of heritability in other reward-related fMRI tasks^49^. While previous studies have linked candidate loci (variants within/near *DRD2, FTO*) to appetitive stimuli processing^18^ and related BOLD networks^19^, we further suggest that our study provides the first evidence for a genetic overlap between the two traits, by demonstrating an association between polygenic risk for adiposity and striatal activation. These observations were robust to potential demographic (age, years of education and socioeconomic status), anthropomorphic (gender and height) and motion (framewise displacement) confounds. We also suggest that the association between BMI and VS BOLD may be observed across a range of rewarding stimuli (such as monetary and appetitive food), consistent with previous reports^6,9^. It is also worth noting that this association was obtained in a sample of young adults (HCP), suggesting that it is unlikely that was a consequence of any metabolic changes or neurodegeneration associated with longstanding obesity.

Recent evidence also suggests pleiotropy between BMI and other complex, polygenic traits such as cognitive function^50,51^, supporting the broader hypothesis of genetic overlap between BMI and dynamic brain systems. The neural response to reward (as measured via VS BOLD) may also be genetically linked to other complex polygenic traits such as psychosis^52^ and positive emotion^47^, suggesting the phenotype's clinical relevance for a spectrum of psychiatric disorders characterised by alterations in reward/hedonic tone. The RPS approach that we used to index an individual's cumulative genetic risk for adiposity has also shown utility in identifying brain structural mechanisms associated with increased risk for obesity^53^, future studies could use the RPS approach to identify specific biological pathways that link obesity-related phenotypes and genetic risk loci.

Although VS BOLD was heritable, one limitation of the study is that the estimates for additive genetic factors influencing VS BOLD were relatively small (H2r = 0.12). This suggests either (a) a limited role for additive genetic variation in the processing of reward stimuli or (b) fMRI methods are more susceptible to noise that structural MR measures of the VS which was moderately heritable, as previously reported^54,55^. Even though we attempted to control for the (genetic) head motion confounding, we also issue caution interpreting the impact of heritable traits that are genetically and phenotypically linked movement confounds, as our movement measure (FD_FSL_) attenuated the observed associations. It is also worth noting that, while we chose to explore BOLD in the VS (to limit comparisons and maximise power^20^), this observation may not be specific to the VS and may apply to the other regions in the appetitive regulatory network as well. There is also the further consideration that the contrast used in the HCP analysis models the receipt of reward, but not the anticipation—another key reward-processing construct that could not be modelled in the current design. There are also between sample discrepancies in participant age and paradigm (monetary and appetitive stimuli), which may limit the generalisation of our findings. We further suggest that the neural networks that support monetary reward and appetitive viewing may also be further modulated by other cognitive networks implicated in the pathophysiology of obesity (such as those that support working memory/executive function^56–58^). Although our study aims to identify causal explanations for the association between BMI and reward-related striatal BOLD, we are aware of the limitations of the cross-sectional design. We also note that we did not have a formal measurement of BMI in the Cardiff sample, although the BMI-RPS was positively associated with sex-adjusted weight, showing evidence for predictive utility. This is a limiting factor due to the complex interplay between obesity and reward processing across the lifespan, where the neural response to reward may be attenuated in middle/older age^59^, which may not be accounted for in the current samples. The impact of elevated BMI across the lifespan may also further confound causal links between genetic risk and appetitive processing, which has not been explored in this study. Furthermore, we do not have the genetic HCP data to identify specific candidate mechanisms/pathways by which the shared genetic influence affects both BMI and reward-related striatal BOLD. Because of these considerations, we suggest that the evidence for a broad genetic overlap between BMI and VS BOLD should be preliminary rather than confirmatory.

In conclusion, this study confirms the presence of a phenotypic and genetic correlation between BMI and reward-related striatal BOLD in young adults. These findings suggest that shared genetic risk factors may explain why individuals who have higher BMI (and risk for obesity) are also more likely to have an elevated striatal reward response. Understanding mechanisms of genetic risk on reward-related striatal BOLD may be instrumental in the prediction, diagnosis and intervention for individuals at risk for obesity.
